# The Telocytes: Ten Years after Their Introduction in the Scientific Literature. An Update on Their Morphology, Distribution, and Potential Roles in the Gut

**DOI:** 10.3390/ijms21124478

**Published:** 2020-06-24

**Authors:** Maria Giuliana Vannucchi

**Affiliations:** Department of Experimental and Clinical Medicine, University of Florence, 50139 Florence, Italy; mariagiuliana.vannucchi@unifi.it

**Keywords:** PDGFRα-positive cells, fibroblast-like cells, interstitial cells of Cajal, myofibroblasts, myoid cells, transmission electron microscopy, immunohistochemistry, nurse cells, differentiation cell

## Abstract

Ten years ago, the term ‘telocyte’ was introduced in the scientific literature to describe a ‘*new*’ cell type described in the connective tissue of several organs by Popescu and Faussone-Pellegrini (2010). Since then, 368 papers containing the term ‘telocyte’ have been published, 261 of them in the last five years. These numbers underscore the growing interest in this cell type in the scientific community and the general acceptance of the name telocyte to indicate this interstitial cell. Most of these studies, while confirming the importance of transmission electron microscopy to identify the telocytes with certainty, highlight the variability of their immune phenotypes. This variability was interpreted as due to (i) the ability of the telocytes to adapt to the different sites in which they reside; (ii) the distinct functions they are likely to perform; and (iii) the existence of telocyte subtypes. In the present paper, an overview of the last 10 years of literature on telocytes located in the gut will be attempted, confining the revision to the morphological findings. A distinct chapter will be dedicated to the recently hypothesized role of the telocytes the intestinal mucosa. Through this review, it will be shown that telocytes, despite their variability, are a unique interstitial cell.

## 1. Introduction

There are many types of cells in the loose connective tissues. Some of them are autochthon, while others migrate in these tissues to perform their functions. Examples of the former case are fibroblasts, fibrocytes, mast cells, and adipocytes. The *fibroblasts* are large-sized cells with few short and thick processes. The oval shaped body carries a clear nucleus containing a large nucleolus. The cytoplasm is basophilic. Under the transmission electron microscope (TEM), fibroblasts show a large Golgi apparatus, extended rough endoplasmic reticulum, and several mitochondria. The membrane is devoid of caveolae and basal lamina. They never form cell-to-cell contacts and are responsible for the extracellular matrix (EM) synthesis. The exact identity and role of the fibrocytes are under debate: are they quiescent fibroblasts or, instead, correspond to the telocytes (TCs) [1]. The *mast cells* are round or oval shaped and contain a central nucleus and numerous basophilic and metachromatic granules enclosing molecules that are able to regulate the exchanges between blood and tissue. Dispersed adipocytes are often seen in the loose connective tissue, especially in large mammals and humans. They are unilocular adipocytes whose large body is filled by a single fat vacuole containing triglycerides. The heterochromatic nucleus is restricted to the periphery, as are the other organelles. Under the TEM, adipocytes show a prominent smooth endoplasmic reticulum and numerous pinocytotic vesicles involved in lipid biosynthesis and transport. These cells are surrounded by a basal lamina and by reticular fibers. Adipocytes are support cells carrying energy store, cushioning, and padding functions.

The migrating cells are the macrophages and other immune cells.

All of the above-mentioned cells are also called interstitial cells (ICs) or stromal cells. All of them originate from the mesenchyma.

The stroma of the gastrointestinal (GI) apparatus contains almost all the above-listed ICs as well as other particular ICs: the interstitial cells of Cajal (ICCs, see Appendix A), the telocytes (TCs), the *myoid cells* (see Appendix C), and, perhaps, the myofibroblasts (Myo, see Appendix B). Apart from ICCs, none of these cells is unique to the GI apparatus. However, their co-existence, their phenotypical similarities and, often, their co-presence in the same layers of the GI wall have brought about misinterpretation, attributing morphological and immunohistochemical properties (and functions) to the wrong cell type. TCs are particularly exposed to these misinterpretations, both because their identification is recent and because their nomenclature is not yet uniform among the research groups.

In the present review, the available morphological data on gut TC will be revised. In particular, this paper focused on the central role of TEM in discriminating among the numerous cell types located in the gut interstitium.

The necessity to correctly identify the single cells and harmonize the nomenclature is fundamental from both the biological point of view and, even more, from the pathological one.

## 2. Telocytes

In 2010, a paper was published [2] where the term telocytes (TCs) was first proposed for a ‘*new*’ type of interstitial cell whose existence had been previously supposed by several researchers referring to it in different ways. Some of them were clearly erroneous (fibroblasts [3] or interstitial cells of Cajal [4]), other appeared too vague (interstitial cells [5]) while others resulted in confusion since they were based on similarities with other stromal cells rather than on TCs own peculiarities (fibroblast-like cells [6] interstitial Cajal-like cells [7,8]).

**Figure 1:**
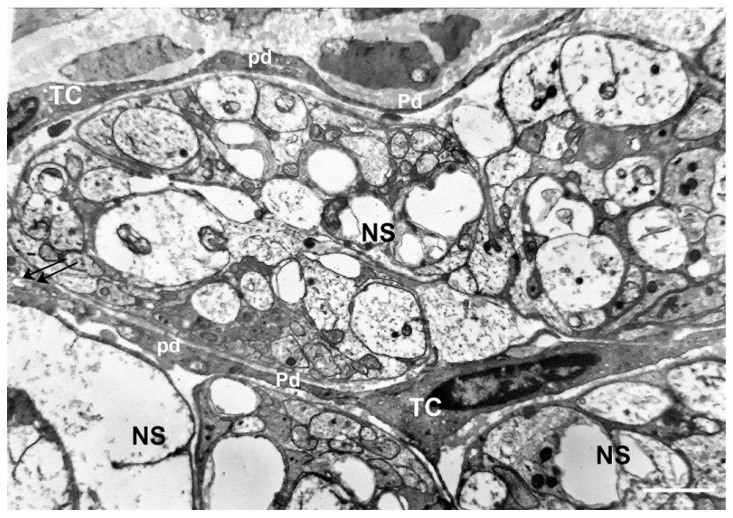
Transmission electron microscope. Human colon. Myenteric plexus. Cells (TC) with a small oval or triangular body and long and thin processes encircling nerve strands (**NS**). Along the processes are knobs containing mitochondria (podoms pd) alternated with thin segments (podomers Pd). Arrows indicate contact areas between the cell processes of two TCs. Bar: 1.3 μm.

**Figure 2:**
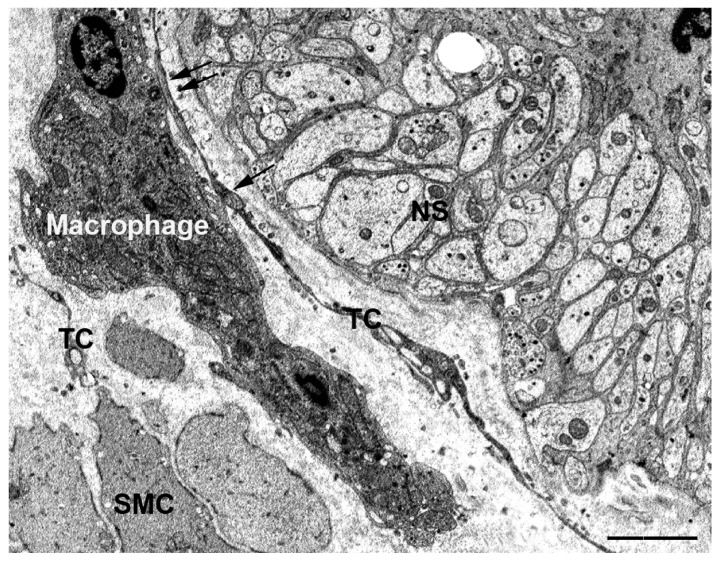
Transmission electron microscope. Human colon. Submucosa border. A large cell (macrophage) makes several cell-to-cell contacts with telocyte (TC) processes. The arrows indicate the contacts. SMC: smooth muscle cells; SN: nerve strand. Bar: 1.4 μM.

**Figure 3:**
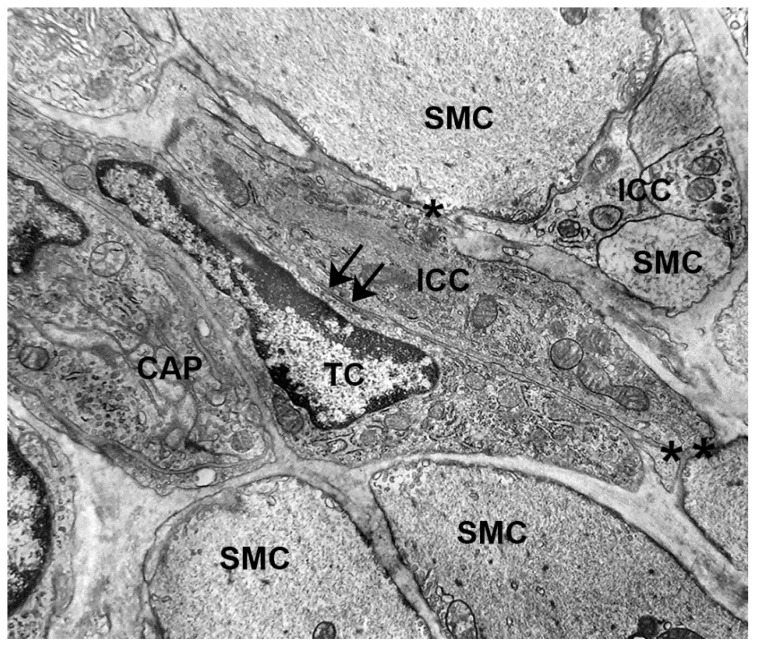
Transmission electron microscope. Mouse stomach. Circular muscle layer. A telocyte (TC) runs parallel to an interstitial cell of Cajal (ICC) and forms a cell-to-cell contact (arrows). The ICC takes several cell-to-cell contacts (asterisks) with the smooth muscle cells (SMC). CAP: capillary. Bar: 0.3 μM.

**Figure 4:**
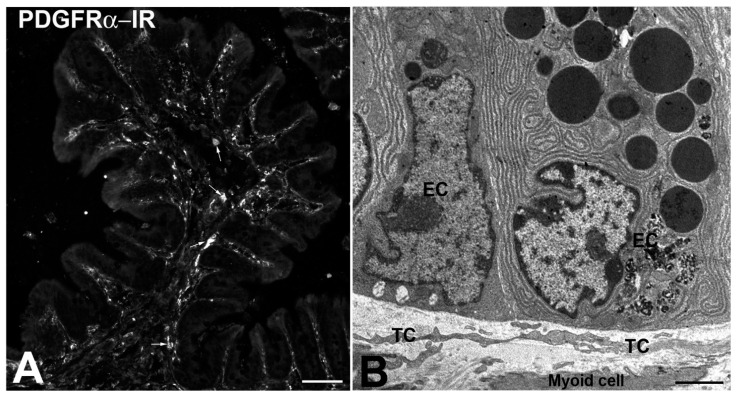
(**A**) PDGFRα-immunoreactivity (IR). Mouse colon. (**A**) Villus. The labeling describes the entire profile of the villus lining the epithelium. (**B**) Transmission electron microscope. Mouse colon. Mucosa. Some TC processes are apposed to the cryptal epithelial cells (EC). Under the TC processes, a myoid cell is present. Bar: A = 40 μM; B = 0.2 μM.

The term TC was proposed based on the cell unique TEM features [2,9]. The ‘ultrastructural hallmark’ of the TCs are the extremely long and thin, dichotomously branched, cytoplasmic processes called Telopodes (Tps). The Tps measure up to 1000 μm in length and between 0.05 and 0.2 μm in thickness and have a moniliform profile characterized by the alternance of thin tracts with dilations. The thin segments are called podomers (average diameter, 75–80 nm) and the dilated regions podoms (average caliber, 250–300 nm). Podoms hold functional units consisting of mitochondria, endoplasmic reticulum, and caveolae. The cell body, measuring between 9 and 15 μm in length, has a variable shape: fusiform/pyriform/triangular, depending on the number of Tps. The nucleus contains condensed heterochromatin; the surrounding cytoplasm is scarce and encompasses few organelles (Figure 1, Figure 2, Figure 3 and Figure 4). Based on these elements, the authors recommended TEM as the best instrument for TC recognition.

Since [2] was published, additional data have been collected on TCs and some differences have been reported both in the ultrastructure and immunohistochemical labelling. Ultrastructural differences are the least evident; in contrast, differences in the TCs’ immune-phenotypes have been found to be significant [1,9,10,11,12,13,14].

To date, the identity of TCs is still being debated and, even in the most recent literature, an overlap between them and other interstitial cells is frequent. Most of the difficulties in giving a definitive uniqueness to TCs depend on two elements: (i) the absence of a selective immune marker; and (ii) the unknown or, only partially known, role(s) these cells play in the organs. Probably, the two elements are related, namely, the TCs express different immune markers according to the role(s) they play. Currently, there are two markers that, although not exclusive for this cell type, are commonly considered reliable to label the TCs, CD34 and PDGFRα.

Starting from these immunohistochemical (IHC) data, some researchers have chosen to call the TCs, ‘PDGFRα-positive cells’, and this definition is often used in scientific reports. However, we consider this nomenclature to be limited for some reasons. First, and most significant, not all the TCs are PDGFRα-positive [10,11]; second, in the GI tract [15], as in other districts [10,11], beyond the endothelium, mast cells also express PDGFRα; and third, identifying a cell with an immune marker is a common practice for the subclasses of a single cell type (i.e., the immune cells). However, in the gut, the use of PDGFRα labelling is recommended since none of the ICs that has a similar shape as, and shares the same territory of the TCs, expresses this receptor. Furthermore, the availability of this reliable (although not selective) labelling has allowed it to be demonstrated that TCs express numerous functional molecules such as ion channels, receptors for different transmitters or hormones, and factors involved in controlling cell proliferation and differentiation.

### 2.1. The Telocytes in the Gut Form Networks

In the gut muscle coat, the TC network runs parallel or even intercalates with that formed by the ICCs [15,16,17,18] so the possibility that TCs and ICCs could be the same cell type was initially considered. However, by using IHC, TEM and immune-TEM techniques, the unique identity of the TCs has been clearly proven [16,17]. These studies showed, by IHC and immune-TEM, that TCs express CD34 [16] and, by IHC, PDGFRα and that they were c-Kit negative [17,18,19,20]; conversely, ICCs that were c-Kit positive expressed none of these two markers (for ICC ultrastructural features, see the related insert). At the myenteric plexus and in the thickness of the muscle layers, TCs and ICCs form three-dimensional (3-D) networks, whereas, at the border between the circular muscle layer and the submucosa, the networks are bi-dimensional (2-D). However, the TC network faces the submucosal border while that of ICCs faces the muscular one [15,16,17,18,19].

Unlike ICCs, TCs are also present in the submucosa where their network envelops the local ganglia, forms tight meshes in the loose connective tissue, embraces the muscularis mucosae, and terminates in the axes of the intestinal villi (mucosal layer) bordering the gastric glands, the intestinal crypts, and the entire profile of the epithelium [17,20]. To note, in the mucosa, the TCs net is 2-D.

In the submucosa, fibroblasts and fibrocytes are also present, but only TCs are organized in the network. In the mucosa, the TC network lines myoid cells, fibroblasts, and, possibly, Myo. However, only TCs are PDGFRα- and CD34-positive [16,17]. It is worth mentioning that PDGFRα/c-Kit double-labelled interstitial cells have been described in the gut (and urinary) mucosa; these cells likely correspond to the mast cells [10,14,15]. Moreover, a series of recent studies, focused on the role of the mesenchymal cells in the development, differentiation, and proliferation of the gut glandular and lining epithelium, has highlighted the role of the underlining mucosal stromal cells. The identification of these cells, however, is not univocal.

In summary, the TCs are always organized in networks and, most likely, this condition is mandatory to perform their functions.

#### The Telocyte Network Is the Scaffold of the Gut Wall

The most obvious function of this network in the gut is to be a scaffold that guarantees mechanical and rheological support to the connective components during peristalsis and maintains the integrity of the ganglia (Figure 1) during bowel movements aimed at digestion or absorption [16,17,21]. This function is particularly evident in the gut submucosa where the TCs form a dense network [15,16,17]. However, several data suggest that the TC scaffold is more than just a mechanical structure. The possibility that this scaffold might be implicated in the regional distribution of other connective cells (i.e., the macrophages) is reliable (see below).

TCs could also have the role of presiding over the 3-D organization and maintenance of the extra-cellular matrix (ECM) usually produced by fibroblasts. Indeed, in adult life, *fibroblasts* and TCs coexist in the interstitium of almost all organs [22] and, sometimes, TCs represent the largest population. In the presence of a pathological loss of TCs, the fibroblasts increase in number and produce a large amount of ECM, which, however, is disorganized and the integrity of the tissues is greatly compromised [23,24]. Finally, data from other organs suggest that TCs may also be able to synthesize the ECM under hormonal stimuli [25,26].

### 2.2. The Telocytes in the Gut Interact with Other Cells

In the gut, TCs interact with a large variety of connective cells by establishing functional communications and cell-to-cell contacts [12,15,16,17,27]. The cell-to-cell contacts are located lengthwise in the long Tps and consist of minute junctions or, more often, of extended apposition of the contiguous plasma membranes that might act either as mechanical cell-to-cell attachments or as sites of intercellular communication [27]. Furthermore, the TCs might functionally interact with the neighbor cells producing exosomes. These are vesicles of different sizes that behave as intercellular shuttles carrying biological signals [9,26]. Through these ways of communication, the TCs interact with isolated cells (i.e., immune cells), with cells organized in networks (ICCs), sometimes forming “mixed networks” with them, or with cells organized in layers (*smooth muscle cells,* SMCs).

#### 2.2.1. The TCs Interact with Isolated Connective Cells

In the stroma of the gut and of many other organs, TCs are often closely apposed to immune cells (plasma cells, lymphocytes, mast cells, eosinophils, and basophils), and in the narrow spaces between them, small exosomes are present. It has been assumed that the TCs have a role in immune-regulation and immune-surveillance. Shortly, the TCs, by exocytosis vesicles, could release soluble chemoattractant molecules along their processes, acting as guides for the immune cells, and either by their contacts or exosomes, present tissue derived antigens to these cells [9,12,14,27].

Although macrophages belong to the immune cell class, they deserve a separate discussion because of the peculiar role attributed to these cells in the muscle coat of the GI apparatus. In fact, recent studies have demonstrated the existence of GI resident *macrophages* that share an anti-inflammatory phenotype [28] and whose differentiation and maintenance are regulated by enteric neurons [29]. These GI macrophages would be directly involved in intestine homeostasis and functions [30,31]. By using TEM, we showed that in human and mouse gut, TCs and macrophages have extended cell-to-cell contacts (Figure 2) and the TCs almost encircle the macrophages with their long and thin processes. These extended areas of contact might guarantee an adequate distribution of the macrophages in the different layers of the gut wall. Furthermore, such extended contacts could represent a macrophage support when the meshes are stretched during the contractile activity. Interestingly, the TCs are often intercalated between the macrophages and the SMCs making contacts with both cell types. This finding is suggestive for an intermediate role of the TCs between these two cell types [32].

#### 2.2.2. The TCs Interact with Cells Organized in Networks

In the gut muscle coat, the TC and ICC networks are parallel and sometimes intermingled, forming cell-to-cell contacts with each other (Figure 3) [15,16,17]. The existence of this spatial and cellular interaction between TCs and ICCs might have more than one significance:

(i) The TCs might be adult stromal mesenchymal cells able to differentiate in ICCs [16,26]. Notably, while ICCs undergo apoptosis with time, their number does not change significantly in aging [33]. However, images of ICCs mitosis were never seen. Thus, the existence of a pool of stem cells (the TCs?) committed to become ICCs when needed is reasonable. The expression of PDGFRα by the TCs reinforces such a hypothesis since the literature data indicate that PDGF/PDGFR signaling plays critical roles in mammalian organogenesis and morphogenesis [34,35].
(ii) ICCs are constantly and often richly innervated and participate in the control of the muscle wall activity as intermediaries of neuronal actions [15,36,37,38]. Although under light microscopy excitatory and inhibitory nerve fibers are often seen in the vicinity of the TCs [19,20,27,39], using TEM, we demonstrated in the GI muscle coat of mice and humans that the nerve endings never established cell-to-cell contacts with TCs [27,32]. Nevertheless, the TCs, through the contacts with the ICCs, might be involved in neurotransmission, possibly contributing to spread the slow waves or amplify the nervous stimulus generated in the ICC [16,19,20].
(iii) TCs and ICC are involved in the SIP (Smooth muscle cells-Interstitial cells of Cajal-PDGFRα-positive cells) syncytium (see below).

#### 2.2.3. The TCs Interact with the Smooth Muscle Cells

The TCs form several types of contact with the SMCs (27). As above-mentioned, the TCs might mediate the macrophage actions on the SMCs, intercalating between one another. Furthermore, the finding that the TCs established cell-to cell contacts with both the SMCs and the ICCs (Figure 3), has brought about the design of an integrated circuit that comprises SMCs, ICCs, and TCs (or PDGFRα-positive cells) responsible for the correct function of the muscle coat. This circuit has been called ‘SIP syncytium’ [39,40].

#### 2.2.4. Are TCs Target of Neural Signals

Interestingly, although synaptic-like contacts between nerve varicosities and TCs were not observed under the TEM, it has been demonstrated that TCs express purine receptors P2Y1 and apamin-sensitive SK3 channels and respond to agonists and antagonists to these receptors [18,19,41,42,43]. Furthermore, the TCs/PDGFRα-positive cells selectively expressed the SK3 channel among all the ICs present in the gut interstitium [44] and significant changes in the functionality of these receptors have been associated with GI diseases [45,46,47]. TCs also contain soluble guanylyl cyclase and it has been speculated that they could function as neural transducers responding to ATP and nitric oxide [19,40].

### 2.3. Telocyte Presence and Role in the Gut Mucosa

The presence and roles of the TCs in the gut mucosa need to be separately treated because of the peculiar function(s) TCs might play at this level and because of the necessity to also clarify the TCs’ identity, nomenclature, and distribution in the mucosa.

The mucosa is made by the columnar epithelium plus the annexed glands and the underlying thin layer of loose connective tissue, the Lamina Propria (LP), which, despite its thinness, hosts nerve fibers, blood and lymphatic vessels, and numerous and assorted cells (immune cells, connective tissue cells, smooth muscle cells). The mucosa is separated from the submucosa by the *muscularis mucosae*.

As reported above, the submucosal TC network continues up to the mucosa and forms an elaborate 2-D structure lining the epithelium in a strategic position for the maintenance of epithelium integrity (intestinal barrier) and functionality (absorption). In the following, the roles hypothesized for the mucosal TCs are reported.

#### 2.3.1. The TCs in the Gut Mucosa Transduce Sensory Signals

In the last decade, the presence of thin and elongated cells organized in networks in the LP of intestinal villi has attracted the attention of researchers. Using fine methodologies, Furuya et al. [48], described, in the small intestine, cells with the above-mentioned features that expressed the P2Y1 receptors. The authors hypothesized that these cells intervened in the transduction of sensory and immune signals and in the maintenance of mucosal homoeostasis. Based on the ‘in vitro’ data, they called these cells fibroblasts. Despite the excellent quality of this paper, the choice to call these cells with an improper term (fibroblasts never form networks in vivo) led to a delay in identifying the ‘*new*’ cell type present in the intestinal LP.

Undeniably, ascertaining the proper identity of each cell type located in the LP is challenging because of the presence of numerous cells in a thin space. Discriminating among them under a light microscope is often difficult, unless a selective marker is available. More recently, Kurahashi et al. [19,20], using PDGFRα labelling in human and mouse colon, described cells with identical distribution, organization, morphology, and immunohistochemical phenotype than those reported in Furuya’s paper. They also reported that the PDGFRα-positive cells expressed several signaling molecules such as purinergic and toll-like receptors, fundamental to maintain intestinal homeostasis [49]. Thus, in agreement with Kurahashi et al. [19,20], it is reasonable to think that the Furuya’s sub-epithelial *fibroblasts* [48] are the TCs (or PDGRFα-positive cells) and that these cells are involved in signal transduction. Intriguingly, the demonstration that cultured *fibroblasts* organize in a network [50] is consistent with the hypothesis of an ontogenic connection between *fibroblasts* and TCs [26]. Moreover, it has also been reported that the same TCs, when cultured, lose some of their properties and acquire those of Myo [19,48]. All this information should alert the researchers in the interpretation of the results if obtained ‘in vivo’ or ‘in vitro’.

#### 2.3.2. The TCs in the Gut Mucosa Are Nurse Cells for Stem Cell Niches

The possibility that TCs could induce cell differentiation and/or preserve the stem cell status of the precursors was primarily considered for the myocardium [49,50,51,52]. Subsequently, a relationship between TCs and stem cells was postulated in many other organs, based on both experimental and clinical observations [23,24,26]. Very recently, convincing data have been collected to demonstrate that the cells of the LP underlining the intestinal epithelium are nurse cells for the cryptal stem cell niches [53] and are likely the TCs [54,55,56,57,58].

All these articles, while confirming previous findings [48,59], add significant information on the mechanisms of action of these cells, on the importance of forming a network along the axes of the villi, and although not univocally, on their identity. While some authors [55,57,58] named these cells TCs and showed their PDGFRα positivity, others [54,56] remained partially ambiguous, naming these cells either as TCs (being PDGFRα-positive) or Myo (being αSMA positive). Several reasons can explain this ambiguity. First, the vicinity and, sometimes, the overlap of the numerous stromal cells present in the thin villus axis; second, the similarity in shape of most of these cells; and third, the sharing of some immune markers (see appendices for Myo and myoid cells). All these elements make it difficult to distinguish one cell from the other under a light microscope.

To correctly recognize the identity of the subepithelial nursing cells, it is useful to mention the results obtained by Kurahashi et al. [19,20] and by us [17]. In their studies, Kurahashi et al. [19,20] showed in mouse and human colon that the stromal cells facing the basal lamina of the epithelium were PDGFRα-positive and αSMA-negative, thus, according to our data [17], they are TCs. Kurahashi’s group also showed that the TCs/PDGFRα-positive cells were surrounded and closely apposed to αSMA-positive cells (myoid cells?) on the connective side; however, these two cell types never shared the two labels. Interestingly, the same authors also reported that the TCs, when cultured, modified their phenotype, and might become αSMA positive. This observation could explain the αSMA positivity reported by Greicius et al. [54] when they isolated and cultured the PDGFRα-positive cells. In parallel to Sanders’s work, we demonstrated in mouse (personal communication [60] (Figure 4A,B) and human colon [17] that all the TCs were PDGFRα- and CD34-positive and αSMA-negative; additionally, the mucosal TCs showed co-labeling only in the cryptal portion of the villi and not at their apex [17].

Differences in the expression of the labeling for the TCs are not a novelty and these differences have been related to the diverse functions the cells might play. For example, Vannucchi and coworkers showed the presence of three subtypes of TCs in human urinary bladder lamina propria based on either IHC or TEM observations and each subtype was supposed to play a proper role [10]. Kurahashi et al. [21] reported that the cells at the bottom of crypts were more sparse and showed lower PDGFRα-positivity, while the cells near the apex of the villi were denser and showed higher PDGFRα labeling intensity. Intriguingly, Shoshkes-Carmel et al. [55], Kondo and Koestner, [57], and Koestner [58], using genetic methodologies, distinguished between the TCs located at the bottom and those at the villus apex in term of function: cryptal TCs would regulate the local cell proliferation; those along the villus axis would mediate cell differentiation. The expression of the functional receptor PDGFRα by the TCs agrees with such roles. In fact, PDGF/PDGFR signaling has been proven to be critical for villous morphogenesis in the gut [61] and the selective PDGFR inhibition suppresses longitudinal smooth muscle differentiation in murine embryonic gut [62].

Establishing the right identity of the nursing cells is fundamental both from the biological and pathological point of view. In the latter case, it is important to consider that in inflammatory, degenerative, and tumor GI diseases, events such as proliferation, differentiation, and apoptosis play a major role. Therefore, the identification of the cells involved in the regulation of these events could be crucial to direct the pharmacological intervention [56,59].

## 3. Conclusions

TCs are ubiquitous and unique cells whose name is highly suggestive of their ultrastructural peculiarities and TEM is the best instrument to identify these cells with certainty.

In the GI apparatus, all the TCs express the CD34, as also confirmed by immune-TEM, and PDGFRα; these two markers are reliable to identify the TCs among those IC that share a similar shape and territory. Furthermore, immunohistochemical findings indicate that TCs neither express c-Kit nor αSMA; nevertheless, attention must be paid when the TCs are cultured in vitro. PDGFRα is the most widely used labeling to identify the TCs and several authors call the TCs “PDGFRα- positive cells”. The main limitation of this nomenclature is that not all the TCs express this marker.

TCs always form networks and this organization is probably essential to carry out the many roles attributed to these IC. Many of these roles are probably common to all locations where TCs have been featured. However, being able to identify them in the intestine is fundamental for a better understanding of the physiology of gastrointestinal functionality and the etio-pathogenetic mechanisms underlying many gut diseases.

In summary:

1. TCs are the mechanical support during gut movements.
2. TCs organize the ECM, compartmentalize, and restrain the other IC (i.e., macrophages or other immune cells) inside their meshes.
3. TCs take several types of cell-to-cell contacts with almost all the other IC and, through the production of exosomes, might interact with all the cells present in the gut wall, neurons included.
4. TCs express receptors and contain molecules involved in the neurotransmission and might participate in the control of the GI functions in terms of absorption and motility.
5. TCs might be mesenchymal stem cells as precursors of the ICCs (and possible of other IC);
6. TCs lining the gut epithelium are likely involved in controlling the proliferation and differentiation of cryptal stem cells behaving as nurse cells.

## Abbreviations

TEM	Transmission electron microscope
TCs	Telocytes
ICs	Interstitial cells
GI	Gastrointestinal
ICCs	Interstitial cells of Cajal
Myo	Myofibroblasts
Tps	Telopodes
IHC	Immunohistochemical/immunohistochemistry
NS	Nerve strands
ECM	Extra-cellular matrix
SMC	Smooth muscle cells
LP	Lamina Propria
IR	Immunoreactivity
PDGFRα	Platelet derived growth factor receptor-α
EC	Epithelial cells

## Appendix A

### The Interstitial Cells of Cajal (ICCs)

*ICCs are the best-known and most studied connective cells of the GI apparatus. They were first described by Ramon y Cajal in 1889 who called them “interstitial neurons” because of their long “neuron-like” cytoplasmic projections as they were identifiable through staining techniques that specifically labelled neurons (e.g., methylene blue or silver impregnation) and because they were located in the interstitium between nerve endings and smooth muscle cells (SMCs) [63]. Several decades later, these cells were reconsidered and through morphological [64,65] and genetic [66] studies, their mesenchymal origin was demonstrated. Immunohistochemical investigations have established that ICCs express the Kit receptor [67,68,69] and their ultrastructural features have been well defined: the ICCs have an oval body containing abundant cytoplasm and a clear nucleus and one nucleolus, two or more processes of variable length that become thinner far from their emergency and are often ramified. The cytoplasm contains a normal sized Golgi apparatus, extended smooth endoplasmic reticulum, many mitochondria distributed everywhere, thin and intermediate filaments, many caveolae, and discontinuous basal lamina. The ICCs make contacts with each other, the SMCs, and nerve endings. Furthermore, either by light or electron microscopy, it was demonstrated that the ICCs always form networks. Finally, a very elaborate ICC organization has been described in the GI tract that has led to the sub-classification of the ICCs in several populations distinct for organ and for the layer in which they are located (for an exhaustive information of the different subtypes of ICCs see [70]). In parallel, physiological studies have proven that ICCs are the pacemakers for intestinal peristalsis since it generates the slow waves [71]; that these cells are intermediate in the enteric neurotransmission [36,72,73]; and that the ICCs located in the deep muscular plexus of the small intestine [36,70,74] and in the thickness of the gastric muscle wall [75] are part of the GI ‘stretch receptor’. Although ICCs originate from the mesenchyma, their strict relation with the neurons starts from the embryo period. Studies in human and animal [76,77] embryos show that in the primitive intestine, the development and differentiation of the mesenchymal cells in ICCs are under the control of the neural crest cells, but also that once the ICCs differentiate, they would stop the neural crest cells’ proliferation, favoring the differentiation in neurons. Interestingly, the expression of the c-Kit receptors on ICCs might constitute a key factor in this differentiative circuit.*

## Appendix B

### Myofibroblasts

*Myofibroblasts (Myo) are known as reparative tissue cells or cells forming the stroma of some solid cancers [78,79]. The presence of Myo in healthy tissue is debated [78,79]. The identification of the Myo using IHC is not very informative. These cells are αSMA-, non-muscle myosin- vimentin-positive, and desmin- and smooth-muscle myosin-negative [78]. Thus, only double labelling allows them to be discriminated from most of stromal cells. In contrast, under TEM, Myo are identifiable with certainty: they show a large oval or spindle shaped body and short and thin processes. The cytoplasm is rich in cisternae of rough endoplasmic reticulum (RER) and mitochondria, contains a well-developed Golgi apparatus, and bundles of strictly packed filaments to form a cell-to-matrix junction with the extracellular adhesion protein, fibronectin. These junctions are called: fibronexuses. These latter features allow the Myo to be definitively discriminated from the fibroblasts. Furthermore, unlike fibroblasts, Myo might contact other cells and form homo- or hetero-cellular networks [10,79,80]. In the human bladder, for example, the interstitial cells located in the upper part of the lamina propria are commonly referred as to Myo. They form a hetero-cellular network with the TCs [14,81,82,83,84,85] and are part of the complex sensory system located in this bladder layer [14,83,84,85].*

*In the gut, the presence of Myo in healthy conditions is more questioned [59,78]. The supposed intestinal Myo would be located under the epithelium [59,78]. However, in papers where Myo were identified under TEM, the specimens investigated were always pathological [78]. When the Myo were searched using immuno-markers, the results were ambiguous since the positive labelling obtained was not specific [54,59]. Furthermore, recent reports have shown that the cells lining the intestinal epithelium are PDGFRα-positive, leading to the question of whether the cells previously called Myo are, in fact, the TCs [17,19,20,54,57].*

## Appendix C

### The Myoid Cells

*In the stroma of the intestinal villi of humans and laboratory animals, there have been described peculiar cells called myoid cells [86,87]. These cells are located very close to the basal surface of the lining epithelium. Through IHC, they resulted in αSMA-positive and cKit-negative with a very thin body often undistinguishable from their thin and ramified processes. Under TEM, they share myoid features such as a spindle shaped body containing bundles of myofilaments, caveolae, and a basal lamina, but also present some peculiarities such as thin, elongated, and interconnected branches oriented with their major axis parallel to the major axis of the villi and numerous, extended cell-to-cell contacts with the epithelial cells. Finally, varicose nerve fibers, rich in synaptic vesicles, are constantly seen near them [87,88]. Functionally, it has been proposed that myoid cells are able to respond to mechanical or locally generated chemical stimuli [89]. In summary, myoid cells are morphologically similar to the TCs, but express αSMA as do the myofibroblasts. Notably, all these three cell types are present in the villous axis.*
